# Genome-wide identification of key enzyme-encoding genes and the catalytic roles of two 2-oxoglutarate-dependent dioxygenase involved in flavonoid biosynthesis in *Cannabis sativa* L.

**DOI:** 10.1186/s12934-022-01933-y

**Published:** 2022-10-15

**Authors:** Xuewen Zhu, Yaolei Mi, Xiangxiao Meng, Yiming Zhang, Weiqiang Chen, Xue Cao, Huihua Wan, Wei Yang, Jun Li, Sifan Wang, Zhichao Xu, Atia Tul Wahab, Shilin Chen, Wei Sun

**Affiliations:** 1 Key Laboratory of Beijing for Identification and Safety Evaluation of Chinese Medicine, Institute of Chinese Materia Medica, China Academy of Chinese Medical Sciences, Beijing, 100070 China; 2 College of Life Science, Northeast Forestry University, Harbin, 150040 China; 3 Center for Molecular Medicine and Drug Research, International Center for Chemical and Biological Sciences, University of Karachi, Karachi, 75270 Pakistan; 4 Institute of Herbgenomics, Chengdu University of Traditional Chinese Medicine, Chengdu, 611137 China

**Keywords:** *Cannabis sativa*, Flavonoid metabolic pathway, flavonol, FLS, Gene family

## Abstract

**Background:**

Flavonoids are necessary for plant growth and resistance to adversity and stress. They are also an essential nutrient for human diet and health. Among the metabolites produced in *Cannabis sativa* (*C. sativa*)*,* phytocannabinoids have undergone extensive research on their structures, biosynthesis, and biological activities. Besides the phytocannabinoids, *C. sativa* is also rich in terpenes, alkaloids, and flavonoids, although little research has been conducted in this area.

**Results:**

In this study, we identified 11 classes of key enzyme-encoding genes, including 56 members involved in the flavonoid biosynthesis in *C. sativa,* from their physical characteristics to their expression patterns. We screened the potentially step-by-step enzymes catalyzing the precursor phenylalanine to the end flavonoids using a conjoin analysis of gene expression with metabolomics from different tissues and chemovars. Flavonol synthase (FLS), belonging to the 2-oxoglutarate-dependent dioxygenase (2-ODD) superfamily, catalyzes the dihydroflavonols to flavonols. In vitro recombinant protein activity analysis revealed that CsFLS2 and CsFLS3 had a dual function in converting naringenin (Nar) to dihydrokaempferol (DHK), as well as dihydroflavonols to flavonols with different substrate preferences. Meanwhile, we found that CsFLS2 produced apigenin (Api) in addition to DHK and kaempferol when Nar was used as the substrate, indicating that CsFLS2 has an evolutionary relationship with *Cannabis* flavone synthase I.

**Conclusions:**

Our study identified key enzyme-encoding genes involved in the biosynthesis of flavonoids in *C. sativa* and highlighted the key CsFLS genes that generate flavonols and their diversified functions in *C. sativa* flavonoid production. This study paves the way for reconstructing the entire pathway for *C. sativa’*s flavonols and cannflavins production in heterologous systems or plant culture, and provides a theoretical foundation for discovering new cannabis-specific flavonoids.

**Supplementary Information:**

The online version contains supplementary material available at 10.1186/s12934-022-01933-y.

## Background

Flavonoids are widely distributed throughout the plant kingdom [1]. They function as copigments in flowers [2] and antibiotics in plant defense responses [3], and act as signal molecules in plant–microbe interactions [4]. They also establish an inevitable link with human diet and health [5] because of their antioxidative [6], anti-inflammatory [7], and strong anticancer activities [8]. Flavonoids are subdivided into six groups, including flavanols, flavanone, flavonols, flavones, anthocyanidins, and isoflavonoids [9]. To date, over 4000 different flavonoids have been identified [10], many of which have been testified to have pharmacological activities, such as catechin, rutin [11], apigenin (Api) [12], liquirigenin [13], and cannflavins [14].

*Cannabis sativa* L. is an annual herb of the Cannabinaceae family, the genera *Cannabis* [15]. *C. sativa* has been historically cultivated and utilized for 6000 years for food, textiles, and medicine [16]. Much attention has been primarily given to major phytocannabinoids, whereas besides cannabinoids, *C. sativa* also produces various non-cannabinoids constituents, including lignanamides, alkaloids, spiroindans, dihydrophenanthrenes, and flavonoids [17]. Flavonoids in *C. sativa* are currently understudied [18]. Over 20 flavonoids have been identified in *C. sativa* [19], belonging mainly to two classes, flavonols (kaempferol (K) and quercetin (Q)) and flavones (Api and luteolin) glycosides and aglycones [20]. Three geranylated/prenylated canniflavones, cannflavin A (geranyl), B (prenyl), and C (geranyl), unique to *C. sativa* (cannflavin A has also been detected in *Mimulus bigelovii*) [21, 22] {Rea, 2019 #19;Rea, 2019 #125}, have exhibited potent anti-inflammatory, anti-leishmania, and antioxidant activities, respectively [17, 23].

Many studies have reported the biosynthesis of the core flavonoid skeleton in medicinal plants [14]. For example, there are two distinct pathways from the root and aerial parts of *Scutellaria baicalensis* [24] responsible for synthesizing flavones [25]. The pathway of flavonoid biosynthesis, particularly the early phenylpropanoid biosynthetic steps in plants, show the commonness. Briefly, phenylalanine is used as the precursor substrate to generate the intermediate metabolite naringenin (Nar) through a series of enzymatic reactions, including phenylalanine ammonia lyase (PAL), cinnamic acid 4-hydroxylase (C4H), *p*-coumaroyl: CoA ligase (4CL), chalcone synthase (CHS), and chalcone isomerase (CHI). Subsequently, Nar is flowed to the biosynthesis of either flavonols such K, Q, and myricetin (M) by flavanone 3-hydroxylase (F3H) and flavonol synthase (FLS) or flavones such as Api, luteolin, and cannflavin A, B, and C in *C. sativa* via the catalytic action of flavone synthase (FNS) and other cytochrome P450 enzymes and transferases.

Two gene-encoding enzymes in the early phenylpropanoid biosynthetic pathway, CsPAL and Cs4CL [26], as well as both candidate O-methyltransferase (CsOMT21) and prenyltransferases (CsPT3) that form cannflavin A, B, or C [14, 21], have been identified in *C. sativa.* However, the key enzyme-encoding genes involved in flavonol biosynthesis, have not been systematically identified in *C. sativa*. FLS, a member of the 2-oxoglutarate and Fe(II)-dependent dioxygenases (2-ODD) superfamily, converts dihydroflavonols to flavonols [27]. In this study, we explored and characterized 56 enzymatic genes throughout the flavonoid biosynthetic pathway from a reference *C. sativa* genome assembly. We also identified potentially encoding candidate genes by combining their expression patterns with flavonoid content in different tissues and chemovars of *C. sativa*. We eventually focused on the *CsFLS2* and *CsFLS3* genes, and found that they retained the conservative function of FLS and exhibited additional enzymatic properties in vitro. These results systematically constitute step-by-step genes involved in the biosynthesis of flavonoids in *C. sativa* and entitle a potential function of CsFLS different from FLS in other higher plants.

## Results

### Identification and expression of genes involved in flavonoid biosynthesis in *C. sativa* and determination of flavonoid content in different cannabis chemovars

**Fig. 1:**
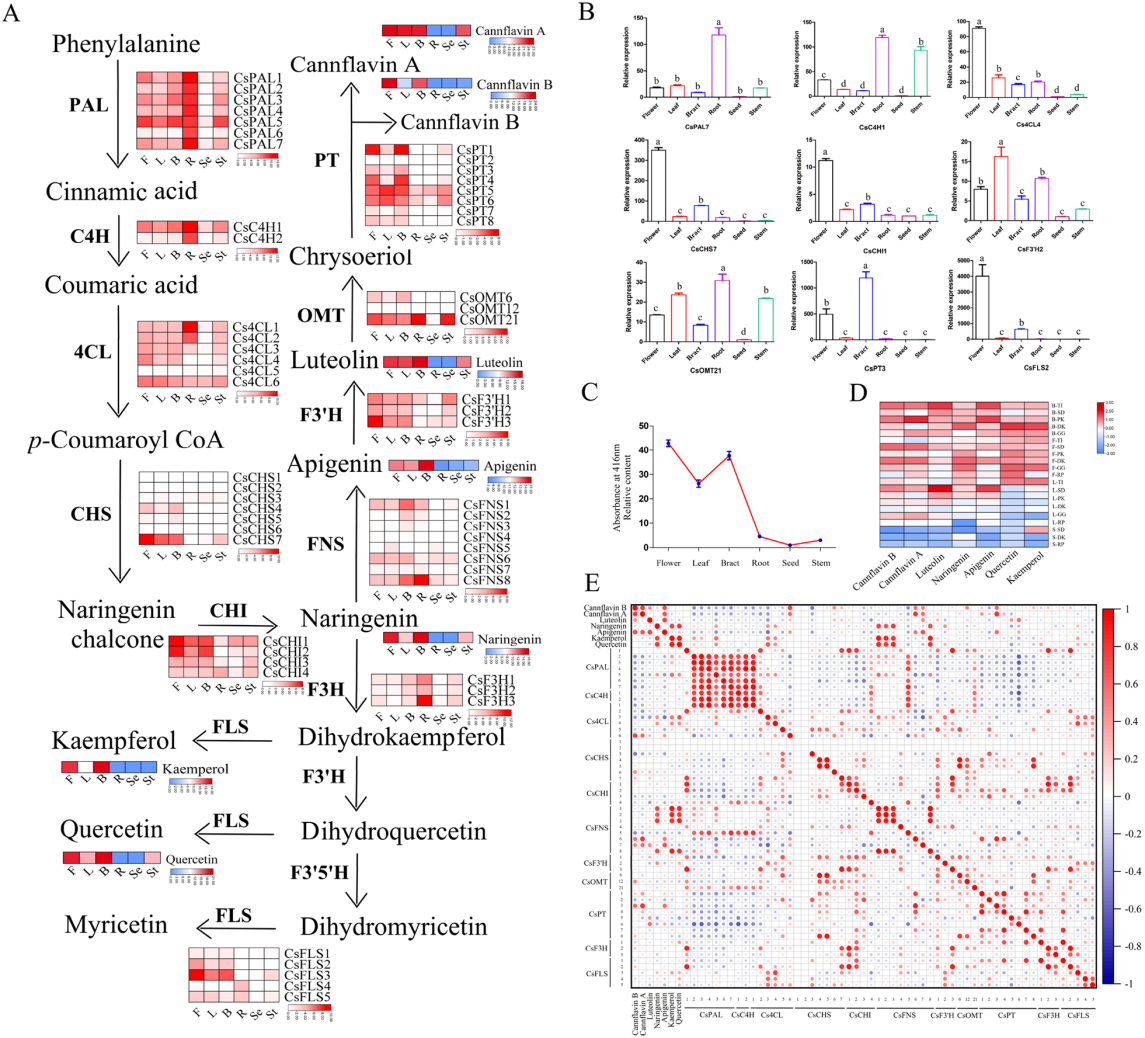
Expression of gene-related flavonoid biosynthesis and flavonoid content in different tissues in *C. sativa*. **A** Schematic representation of gene expression in the flavonoid biosynthesis pathway and accumulation of flavonoids in six tissues of DiKu. Data of the transcriptional level was present by log2 (FPKM + 1). The content of flavonoids was presented by the average of peak area. B: Bract, F: Flower, L: Leaf, St: Stem, Se: Seed, R: Root. PAL: phenylalanine ammonia lyase, C4H: cinnamate 4-hydroxylase, 4CL: 4-coumaric acid: CoA ligase, CHS: chalcone synthase; CHI: chalcone isomerase, F3H: flavanone 3-hydroxylase, FNS: flavone synthase; F3′H: flavonoid 3′-hydroxylase, F3′5′H: flavonoid 3′5′-hydroxylase, FLS: flavonol synthase, OMT: O-methyltransferase, and PT: prenyltransferase. **B** Relative expression of selected genes in different tissues of *C. sativa.* n = 3. Different letter indicates significance (p < 0.05). **C** Accumulation of major flavonoids of *C. sativa.* in different tissues of different chemovars. Data are presented as the average of the peak area.TI: Terra Italia, SD: Swiss Dream, PK: Pain killer, GG: Gorilla Glue, RP: Red Pure, and DK: Dinamed Kush. **D** Variation in total flavonoids in different tissues of *C. sativa.* Results were Mean ± SE (n = 3). **E** Correlation analysis of the transcription abundance of genes participating in the flavonol biosynthetic pathway with contents of cannflavin A, cannflavin B, luteolin, naringenin, apigenin, kaempferol, and quercetin in *C. sativa*

To identify the key structural genes related to flavonoid biosynthesis in *C. sativa*, we screened and explored 56 enzyme genes involved in flavonoid biosynthesis, including seven *CsPALs*, two *CsC4Hs*, six *Cs4CLs*, seven *CsCHSs*, four *CsCHI*s, eight *CsFNSs*, three *CsF3'Hs*, three *CsF3Hs*, five *CsFLSs*, three *CsOMTs*, and eight *CsPTs* using the BLASTP search and SwissProt database. The physical characteristics, including the amino acid (aa) length, molecular protein weight, isoelectric point, and their predicted subcellular localization, were also investigated (Additional file 1: Table S1). These proteins were located in different organelles, indicating that flavonoid biosynthesis in hemp is a complex and synergetic process. The expression patterns of these enzyme genes (Fig. 1A) were investigated based on the RNA-seq (RNA-sequencing) data of six different tissues (flower, bract, seed, root, leaf, and stem) of DiKu. Early genes (*PAL, C4H,* and *4CL*) were highly expressed in root, flower, and bract. The *CsCHS*, *CsCHI*, *CsFNS*, *CsF3'H*, *CsOMT*, *CsPT,* and *CsFLS* gene families were mostly expressed in flowers, bracts, and leaves, while they showed relatively low transcriptional levels in roots, seeds, and stems, which were consistent with the distribution of flavonoids, including Nar, K, Q, Api, and cannflavins A and B. The candidate genes, including *CsPAL7*, *CsC4H1*, *Cs4CL4*, *CsCHS9*, *CsCHI1*, *CsOMT21*, *CsPT3*, and *CsFLS2,* were used to perform qRT-PCR (Fig. 1B and Additional file 2: Table S2), where they were largely consistent with the transcriptome data.

We also determined the content of total flavonoids in different tissues of DiKu (Fig. 1C). The total flavonoids were primarily distributed in flowers, bracts, and leaves, and they were hardly detected in seeds. Meanwhile, the content of different flavonoids in the six chemovars was detected (Fig. 1D). Data of the undetected flavonoids like M and its derivatives were not shown. Generally, flavonoids are inclined to be reserved in flowers and bracts compared with leaves and stems regardless of chemovars, although the content of different flavonoids varied in chemovars.

To characterize key genes involved in flavonoid biosynthesis, correlation analysis was performed based on the expression of key enzyme genes and the flavonoid content in different tissues of six different chemovars (Fig. 1E). Thereinto, *CsPAL2–3* and *5*, *Cs4CL3–4*, *CsFNS1*–*3*, *CsFNS8*, *CsF3’H2*, *CsF3H1,* and *CsFLS1* and *3–5* showed a relatively high correlation with flavanone and flavonols, whereas *Cs4CL6*, *CsCHS4–7*, *CsCHI1*, *CsFNS6–7*, *CsF3’H1*, *CsOMT12* and *21*, *CsPT1* and *3–6*, *CsF3H2,* and *CsFLS2* correlated highly with flavone accumulation in *C. Sativa.*

### Identification of *CsFLS *orthologs and characterization of *CsFLS* genes and their encoding enzymes

FLS is the key enzyme that catalyzes dihydroflavonols to form flavonols [28]. Here, five potential *CsFLS* that may perform the function described above were investigated depending on the correlation analysis and gene expression. *CsFLS* genes were unevenly distributed on three chromosomes (Chr3, Chr5, and Chr7), where *CsFLS2* and *CsFLS3* were located at Chr3 and Chr5, respectively, while *CsFLS4* and *CsFLS5* were presented on Chr7 in tandem duplication (Additional file 3: Fig. S1A). The structure of the five *CsFLS* genes was investigated in that they contained one to three introns, and the whole gene spanned from 1600 bp to 9000 bp. Meanwhile, the length of the protein encoded by *CsFLS* genes ranged from 332 to 364 aa, and four motifs were highly conserved in CsFLS proteins (Additional file 3: Fig. S1B). The predicted protein secondary structure showed that the random coiling consisting of 40.95% to 57.22% of the protein, α-helix represented 32.49% to 35.01%, and the extended strand (16.30–18.40%) and β-turn (4.42–6.02%) accounted for less of the whole secondary structure (Additional file 4: Table S3).

**Fig. 2:**
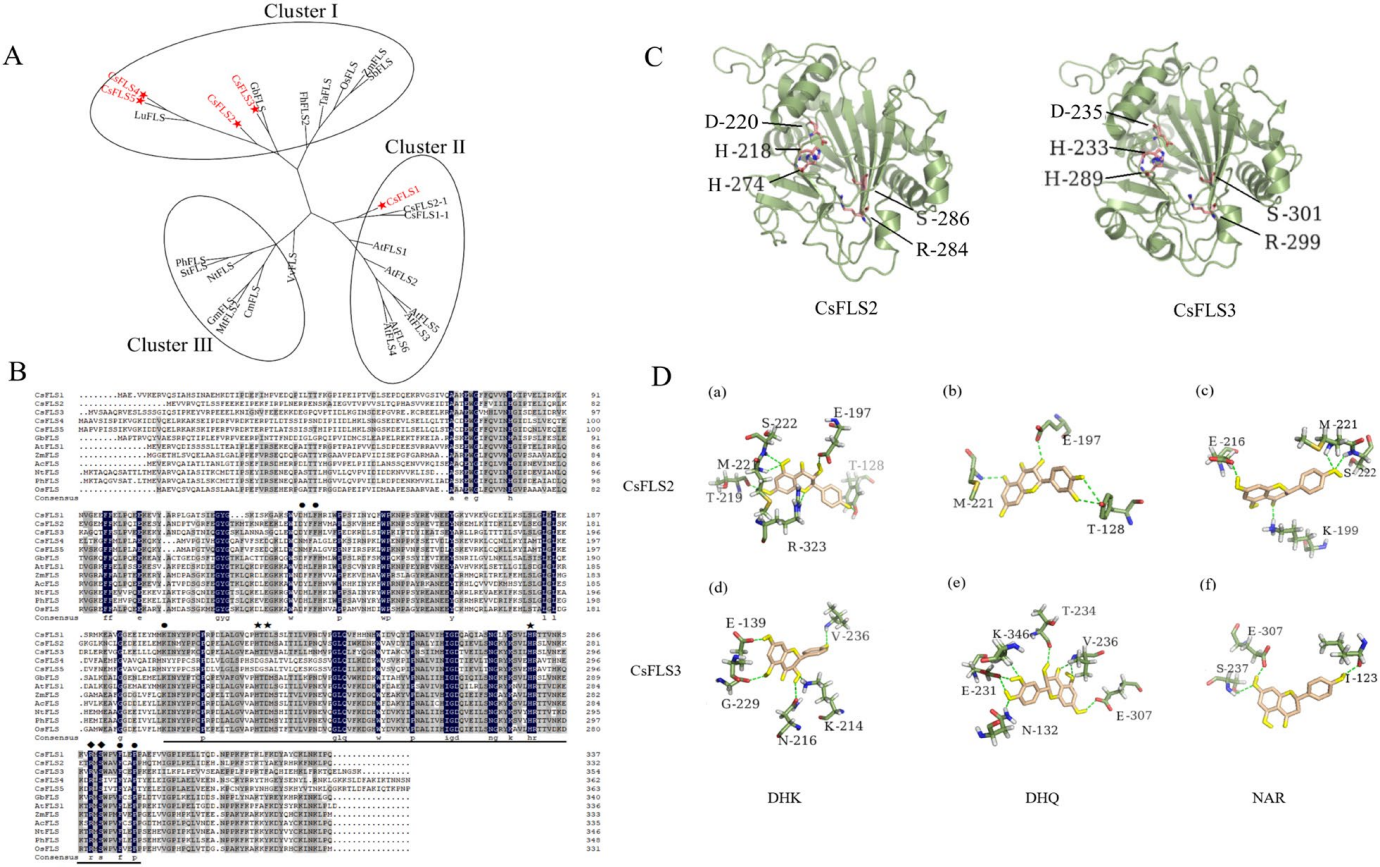
Catalytic characteristics of CsFLS. **A** Phylogenic analysis of CsFLS. The neighbor-joining method was used to construct this tree with 1000 replicate bootstraps using MEGA6.0. The CsFLSs characterized in this work are marked with red stars. Accession numbers of proteins from other plants are shown in Additional file 5. Table S4. **B** Sequence alignment of CsFLSs with functionally verified FLSs in Apiaceae. The proposed residues involved in binding the dihydroquercetin substrate are marked with circles. The conserved domain of 2-ODD was underlined. Inferred amino acids for binding the ferrous iron and 2-oxoglutarate (2-OG) were marked with pentagram and lozenge, respectively. **C** Predicted three-dimensional structures of CsFLS2 and CsFLS3. CsFLS2 binding sites for Fe^2+^ and 2-OG were H218, D220, and H274, and R284 and S286, respectively. Meanwhile, H233, D235, and H289, as well as R299 and S301 are the Fe^2+^ and 2-OG binding sites of CsFLS3, respectively. **D** Prediction of dihydrokaempferol (DHK), dihydroquercetin (DHQ), and naringenin (Nar) interacted with modeling CsFLS2 (**a**–**c**) and CsFLS3 (**d**–**f**) by docking simulation

FLS belongs to the superfamily of 2-ODDs, and is broadly found in flowering plants regardless of monocots or dicots [29]. To identify orthologous FLS proteins in hemp, we performed a phylogenetic analysis of 22 FLSs in 16 different plant species (Fig. 2A and Additional file 5: Table S4). CsFLS proteins were classified into three clades, where CsFLS2–5 were clustered with *Ginkgo biloba* and *linum usitatissimum*, while CsFLS1 was grouped with FLSs of *Citrus sinensis* and *Arabidopsis.* To further analyze the differences in CsFLS at the amino acid sequence level, we conducted a sequence alignment with functionalized FLSs in other seven plant species. CsFLS1–3 had the conserved domian of 2-ODDs with binding sites of the Fe^2+^ and 2-oxoglutarate (2-OG) (Fig. 2B and C) [30], as well as the predicted active sites to bond to dihydroquercetin like other reported FLSs [34]. Nevertheless, CsFLS4–5 lacked the binding sites of Fe^2+^ (H233D and D235S) and dihydroquercetin (K214N and F146A). We also established a model for CsFLS2 (Fig. 2D, a–c) and CsFLS3 (Fig. 2D, d–f) docking to their potential substrates, dihydroflavonol (DHK and DHQ) and dihydroflavone (Nar), respectively. The results showed that hydrogen bonds formed between CsFLS2 and these three substrates (e.g., M221, S222, and E197) were close to the Fe^2+^ and substrate binding sites. Similarly, hydrogen bonds formed from V236, S237, and E307 to CsFLS3 may help the substrates bind more tightly to enzymes.

### Cloning and analysis of the catalytic activity of CsFLS

To analyze the catalytic activity of CsFLS2 and CsFLS3, each gene’s full open reading frame was cloned into the pET28a vector. Recombinant CsFLS2 and CsFLS3 proteins were independently expressed in *Escherichia coil* BL21 (DE3) strain as N/C-terminal proteins fusion with two His-6 tags. Purified proteins were verified using Western blotting at approximately 45 kDa, which was consistent with the predicted molecular weights of 43.86 kDa for CsFLS2 and 45.65 kDa for CsFLS3 (Additional file 6: Fig. S2).

**Fig. 3:**
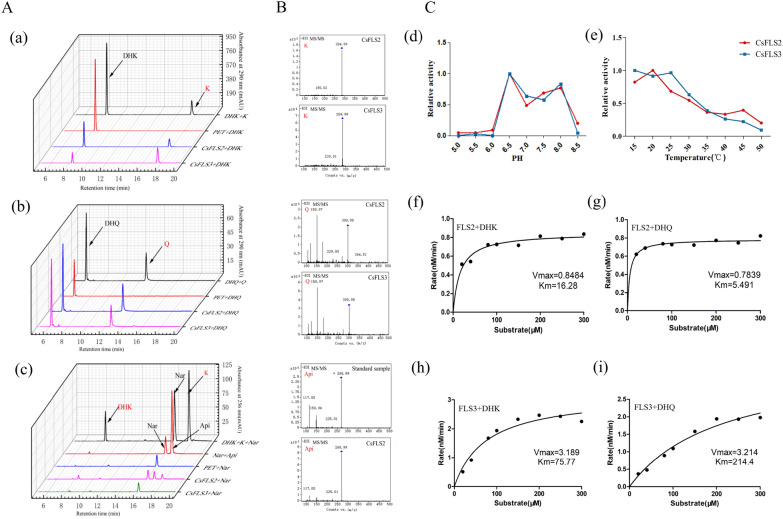
Enzyme activity of CsFLS2 and CsFLS3. **A** HPLC chromatogram of dihydroflavonols (**a** and **b**) and flavanone (**c**) catalyzed by recombinant CsFLS2 and CsFLS3 in vitro. **B** Validation of catalytic products using Q-TOF. **C** Analysis of catalytic activity and preference of CsFLS substrates. (**d** and **e**) The activity of CsFLS2 and CsFLS3 proteins was determined at various temperatures (15–50 ℃) and pH (5.0–8.5). (**f**–**i**) Michaelis–Menten plots for both dihydroflavonols of recombinant CsFLS2 and CsFLS3 enzymes. K, Kaempferol; Q, Quercetin; DHQ, dihydroquercetin; DHK, dihydrokaempferol; Nar, Naringenin; Api, Apigenin

Both CsFLS2 and CsFLS3 converted dihydroflavonols (DHQ and DHK) to flavonols (Q and K), respectively (Fig. 3A, a and b). Additionally, when Nar was used as the substrate, we detected the Nar, DHK, and K in the products. This indicated that CsFLS2 and CsFLS3 had an additional F3H hydroxylation function, which can catalyze the 3-position hydroxylation of Nar to produce DHK (Fig. 3A, c). These data were further verified using LC–MS/MS (Fig. 3B). Interestingly, in addition to DHK and K, another peak occurred in the reaction product of CsFLS2 catalyzing Nar, and the retention time on HPLC, as well as LC–MS/MS, was consistent with the corresponding parameters of the Api standard (Fig. 3B). To better understand this phenomenon, we performed a phylogenetic analysis of 68 genes of DOXC 28/47 subgroups (including *F3H*, *FLS*, *ANS*, and *FNS I*) of the 2-ODD superfamily in 25 different plants with CsFLS2 and CsFLS3 [31] (Additional file 7: Fig. S3A). We subsequently compared the amino acid alignment of CsFLS2 and CsFLS3 with five Apiaceae FNS I in different plant species (Additional file 7. Fig. S3B). The active sites of FNS I consisted of seven amino acid residues [32], of which CsFLS2 possessed three, while CsFLS3 had only one. Together, these data indicated the promiscuous function of the ancestral forms of 2-ODD enzymes and more expansive substrate selectivity [33].

To investigate the activity and preference of CsFLS2 and CsFLS3 with different substrates, the optimum pH and temperature of the enzymatic reactions were first determined using DHK as the substrate. The optimal pH for CsFLS2 and CsFLS3 was pH 6.5, with the highest catalytic activity at 20 ℃ and 15 ℃, respectively (Fig. 3C, d and e). Detailed kinetic studies conducted at the optimum and kinetic parameters were calculated using nonlinear regression analysis with Michaelis–Menten plots [34]. The Michaelis constant (*K*_*m*_) of DHK and DHQ for CsFLS2 was 16.28 µM and 5.49 µM, respectively, indicating that CsFLS2 had a higher affinity for DHQ than DHK (Fig. 3C, f and g). Conversely, CsFLS3 had a larger affinity for DHK than DHQ, with the *K*_*m*_ of 75.77 μM and 214.4 μM, respectively (Fig. 3C, h and i). Therefore, the catalytic function of CsFLS2 and CsFLS3 may be complementary and substrate-selective in hemp.

## Discussion

Flavonoids are important signal metabolites that keep plants resistant to stress and promote the human diet and health needs [35]. The core skeleton of the flavonoid biosynthetic pathway has been well studied in several flowering plants. However, a systematic gene profile involved in flavonoid biosynthesis in *C. sativa* has not yet been investigated. Indeed, integrating genome, transcriptome, and metabolome has been a highly efficient strategy to elucidate the metabolite biosynthetic and regulatory genes. The cascade of genes was proposed by combining the expression of 56 genes from 11 classes of candidates in the flavonoid pathway from different tissues of *C. sativa* with the metabolic detection of flavonoids (Fig. 1A). Teresa et al. [26] predicted two structural genes, *CsPAL* (KC970300) and *Cs4CL* (KC970301) in *C. sativa*, by searching expressed sequence tags against homologous sequences from other plants, which were named *CsPAL4* and *Cs4CL1* in this study*.* Nonetheless, both genes showed negative correlations with the content of detected flavonoids (Fig. 1E). Conversely, we speculated that *CsPAL2–3* and *5*, as well as *Cs4CL3–4* and* 6* participated positively in flavonoid generation in all *CsPAL* and *Cs4CL* genes identified in our study. The distribution of flavonoids has been investigated in different organs in *C. sativa,* and it was reported that they are undetectable in roots and seeds. Likely due to the improved detection methods and techniques, we detected tiny amounts of flavonoids regardless of flavonol, flavone, or total flavonoids, in roots and seeds [26, 36] (Fig. 1A and C). Meanwhile, downstream genes in the flavonoid biosynthetic pathway are barely expressed in both tissues, reducing flavonoids. Interestingly, genes involved in early phenylpropanoid biosynthetic steps to form the intermediate *p*-Coumaroyl CoA showed high expression in roots, indicating that unknown phenolic acid compounds may be present in roots. Additionally, flavonoids were abundant in flowers, bracts, and leaves in all chemovars of *C. sativa* in this study but scanty in stems (Fig. 1D), signifying *C. sativa* as a versatile plant with different metabolites’ accumulation in different organs. *CsOMT* methylated the 3’-O position of luteolin to form chrysoeriol, which *CsPT* further catalyzed to add a geranyl or a prenyl group to form cannflavin A, B, or C [14]. Kevin et al. [18] identified *CsOMT21* and *CsPT3* based on homology and phylogenetic analysis from a draft *C. sativa* genome assembly, supporting our correlation analysis (Fig. 1E).

FLS as a key enzyme controlling the flavonol flux, has been characterized in numerous plant species. Multiple *FLS* genes are always present in the plants [37]. *Arabidopsis* contains six FLS-encoding genes, only two of which showed flavonoid activities [38]. In this study, five *CsFLS* genes were identified from a reference *C. sativa* genome. They were distributed irregularly, not in the tandem repetition on different chromosomes (Additional file 3: Fig. S1), and divided into groups from the phylogenetic tree (Fig. 2A), suggesting that the *CsFLS* genes have diverged with different functions. *CsFLSs* belonging to the 2-ODD gene superfamily possess two highly conserved binding sites, the 2-OG binding site (Arg-X-Ser) and the Fe^2+^ binding site (His-Asp-His) [28]. Unlike *CsFLS1–3,* which had both active sites, *CsFLS4*–*5* retained the binding sites of 2-OG but lost those of Fe^2+^ (Fig. 2B), implying that *CsFLS4*–*5* were involved as complementary genes or differentiated other functions. The primitive function of FLS converted dihydroflavonols to flavonols, while a single FLS with a bifunctional property forming DHK by catalyzing the 3-hydroxylation of Nar has been identified in plants like *Morella rubra* [32], *G. biloba* [39], and *C. sinensis* [40]. *CsFLS2* and *3* were verified to be bifunctional enzyme-coding genes using recombinant protein activity analysis in vitro (Fig. 3A and B) but with different enzyme catalytic efficiency (Fig. 3C). Chua et al. [41] found that the mutation of His 132 and Gln 295 significantly reduced the catalytic ability of AtFLS to DHQ in *A. thaliana*. CsFLS2 differed from CsFLS3 with Ala (A) rather than Gln (Q) at the same Gln 295 site in *A. thaliana*, explaining why CsFLS2 has less catalytic efficiency for DHQ than CsFLS3 (Fig. 3C) [42]. Besides flow to flavonol, CsFLS2 also produced Api (a flavone) by bringing in a double bond between the C2 and C3 positions in the B ring of Nar like a FNSI. Therefore, we performed a sequence alignment to investigate the relationship between CsFLS and FNS I. CSFLS2 retained a portion of the FNSI active sites, but whether this was the primary reason remains unknown. The plant 2-ODD superfamily was divided into three large clusters including DOXA, DOXB and DOXC, where DOXC is involved in the biosynthesis of colorful flavonoids and other secondary metabolites [31]. The 2ODD genes involved in flavonoid metabolism were classified into two distinct clades: DOXC28 and DOXC47. A phylogenetic analysis among CsFLSs and 28/47 DOXC subgroup members from 2-ODD superfamily was conducted. Unsurprisingly, CsFLS3 related closely to anthocyanidin synthase (ANS) (Additional file 7: Fig. S3), which brought into correspondence with the previous study that recombinant ANS could perform the FLS activity [42]. Different gene expression pattern mediates their functions of genes. In tomato, the duplication of *SlDMR6* (*Solanum lycopersicum Downy MILDEW RESISTANCE 6*), which belongs to the superfamily of 2-ODDs, lead to different expression pattern and subsequent subfunctionalization, where SlDMR6-1 exerted roles in pathogen infection, while SlDMR6-2 balanced salicylic acid levels in flowers and fruits [43]. In hemp, CsFLS2 and CsFLS3 were both absent in roots, while CsFLS3 was observed abundantly in Diku flowers, bracts, and leaves, as well as slightly in stem and seeds. Nevertheless, CsFLS2 had a different expression pattern without expresion in stem and seeds (Fig. 1A). These suggested both FLSs might have different regulatory elements and functional differentiation. Interestingly, cis-acting element analysis of both *CsFLS* promoters showed that potentially regulatory mechanism differed between them. The promoter of *CsFLS2* had defense and stress responsiveness elements, while CsFLS3 possessed specific elements response for low-temperature, salicylic acid and Methyl jasmonate (Additional file 9: Fig. S4). Overall, FLS duplication during evolution resulted in the functional divergency in terms of gene and protein structure, as well as gene expression pattern, which might be responsible for different stresses.

**Fig.4:**
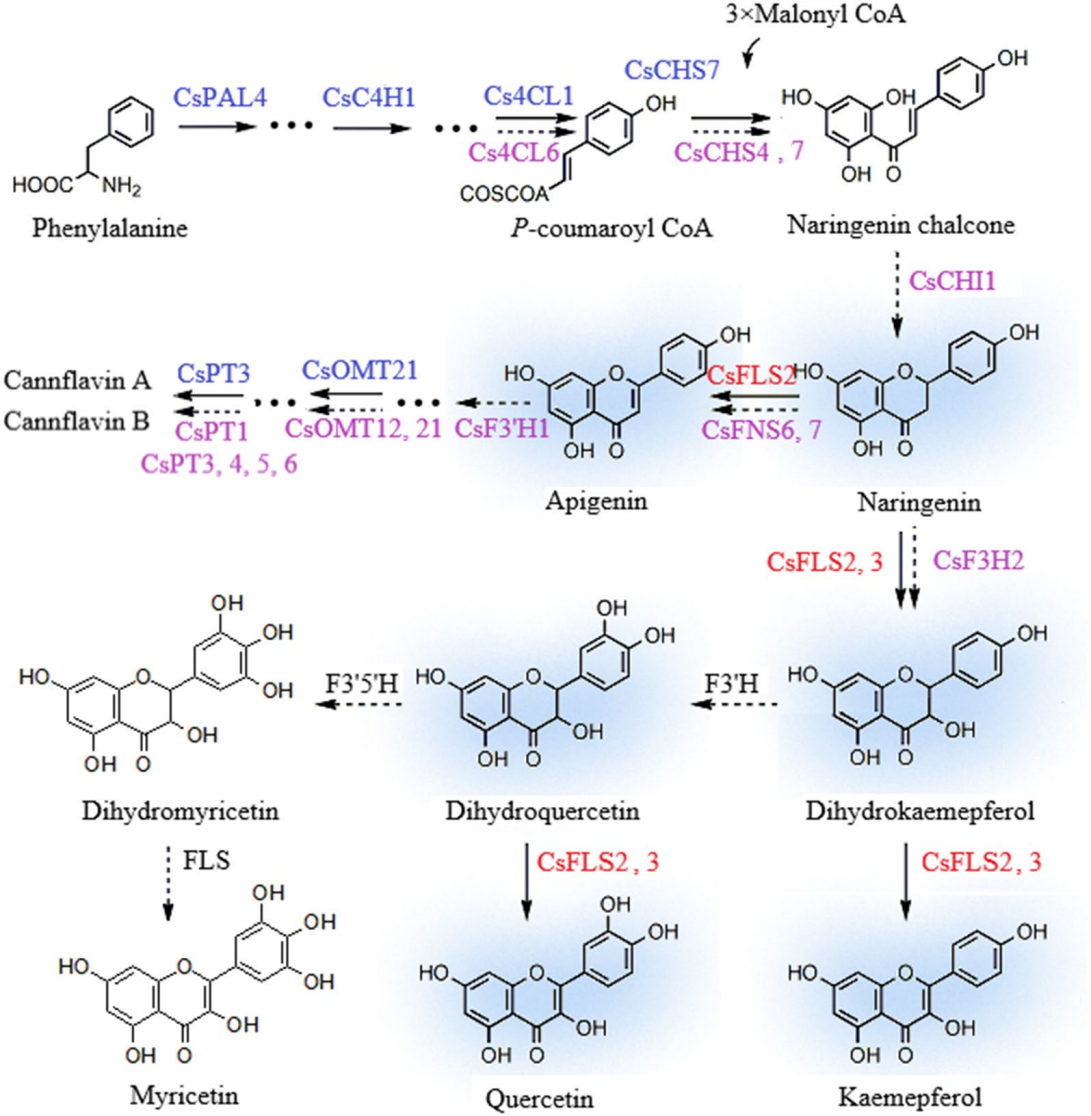
Proposed model of flavonoid metabolism in *C. sativa.* Naringenin (Nar) is converted to dihydrokaempferol (DHK) and keamepferol (K) by CsFLS 2 and 3. Meanwhile, Nar is catalyzed to apigenin (Api) by CsFLS2. CsFLS2 and 3 convert dihydroflavonols to flavonols. Genes highlighted in red are verified in this study. Genes in purple mean that these genes are predicted to exert catalytic action during the steps in this study. Genes marked in blue have been identified in previous studies. Dashed lines represent genes have not yet identified via enzymatic reaction

The accumulation of flavonols, such as quercetin and kaempferol, and flavanone varied in tissue specificity and chemovars of *C. sativa*, suggesting the involvement of *CsFLSs.* Coincidentally, FLS and other flavonoid-related structural genes responded positively to different environmental variations [44, 45] and developmental growth stages [37]. Hence, demonstrating the flavonoid gene cascade (Fig. 4) and understanding how to manage stress-induced flavonoids will be essential for developing environmentally resilient *C. sativa* plants and the biosynthesis of a substantial amount of bioactive flavonoids for downstream usage. Additionally, it was reported that flavonoids, synergistic with other non-phytocannabinoids compounds, exerts the entourage effect of boosting the bioactivities of phytocannabinoids [14]. Therefore, elucidating the mechanism of flavonoid production will have significance in the accumulation of phytocannabinoids and other non-phytocannabinoid compounds and the development of therapeutics in *C. sativa.*

## Conclusions

This study proposes step-by-step potential enzymes involved in the flavonoid biosynthetic pathway in *C. sativa* via a combination of transcriptomics and metabolomics of tissues of different chemovars (Fig. 4). Among these identified genes, *CsFLS2* and *3* encoding enzymes were verified to be the key enzymes controlling flavonol flux via the activity analysis of recombinant proteins. Besides the primitive function of FLS converting dihydroflavonol to flavonol, CsFLS2, with versatile properties, can directly orient the production of both flavonol and flavone. Therefore, this study paves the way for reconstructing the entire pathway in heterologous systems or plant culture to yield flavonols and cannflavins in *C. sativa*. Additionally, this study provides a theoretical foundation for discovering new cannabis-specific flavonoids.

## Methods

### Plant materials and growth conditions

High-CBD chemovars, Dinamed Kush (DiKu), is a feminized plant, crossing Purple Kush and Dinamed Autoflowering CBD. DiKu and other commercial chemovars, Terra Italia, Swiss Dream, Pain killer, Gorilla Glue, and Red Pure, were grown in controlled growth chambers at 25 ℃ with 16:8 (light: dark) photoperiod in Yunnan Dali, China. The samples used in this study, including flowers, bracts, leaves, stems, and roots, were collected 20 days post-flowering (DPF). Once collected, samples were frozen in liquid nitrogen and stored at − 80 ℃ for further use.

### Data sources

The reference genome and annotation files of *C. sativa* (Accession number: GCA_900626175.1) were obtained from NCBI (National Center of Biotechnology Information) [46]. The transcriptomes of data for six different tissues of DiKu were available at NCBI (Accession No.: bract: SMAN16122880-SAMN16122882; stem: SAMN16122883-SAMN16122885; flower: SAMN16122886-SAMN16122888; leaf: SAMN16122889-SAMN16122891).

### Identification and characterization of genes related to the flavonoid metabolic pathway

Protein sequences of PAL, C4H, 4CL, CHS, CHI, FNS, F3′H, OMT, PT, F3H, and FLS from *Arabidopsis thaliana* were downloaded from the Uniport [47] and homology matching was conducted by using BLASTP search and ‘Blast Several Sequences to a Big Database’ in TBtools with the setting of E-value < 10^–5^ and removing redundant sequences. Subsequently, the conserved domain (CD) search tool of NCBI was used to screen out those which did not have complete conserved domains to obtain the members of 11 classes in *C. sativa*. Molecular weights and isoelectric points were predicted using the ExPASy-ProSite website [48]. Subcellular localization predictions were performed using the softberry website [49].

### RNA isolation and first-strand cDNA synthesis

Total RNA was extracted from the DiKu tissues of 20 DPF, with three biological replicates using RNAprep Pure (DP441, Tiangen, Beijing) according to the manufacturer’s instruction. First-strand cDNA was subsequently synthesized using StarLighter Script RT all-in-one Mix (FS-P1001, Foreverstar, Beijing, CN).

### Transcriptomic data analysis and Gene expression pattern analysis

RNA sample sequencing was conducted via Illumina and PackBio sequencing platforms. After trimming the redundancy reads, clean reads were mapped to the reference *C. sativa* genome sequence by employing HISAT2 tools, where 31,170 genes referred to 41,553 transcripts were annotated. Gene expression levels were estimated as fragments per kilobase of transcript per million fragments mapped (FPKM), of which log 2 (FPKM + 1) was assessed using TBtools [50] to represent gene expression from different tissues of DiKu (flowers, leaves, bracts, roots, seeds, and stems).

### Quantitative real-time PCR validation

qRT–PCR was performed on Rotor-Gene Q (QIAGEN, Germany) using a StarLighter SYBR Green qPCR Mix kit (FS-Q1002, Foreverstar, Beijing, CN). The program was set as 95 °C for five minutes, 95 ℃ for 30 s, 60 ℃ for 20 s, and 72 ℃ for 15 s for 40 cycles. Each sample was repeated at least thrice, and the data were analyzed using the 2^−(ΔΔCt)^ method [51] with *EF1α* [52] as the reference gene. The relevant primers are shown in Additional file 2: Table S2.

### Analysis of flavonoid content in different tissues of *C. sativa*

The collected samples were lyophilized and then ground into power. Next, 100 mg of the ground sample was exposed to 1 ml of 70% methanol, sonicated at room temperature for 30 min, and placed at 4 °C overnight. The supernatant was retained after a 12,000 rpm centrifugation for 15 min. All samples were extracted twice following the steps above, and the mixed supernatant was filtered through a 0.22-μm organic membrane for total flavonoid content determination and LC–MS/MS analysis.

120 μl of diluted solution (in an appropriate proportion) and 60-ul NaNO_3_ (5%) were mixed and stayed for six minutes with an addition of 60-μl Al(NO_3_)_3_ (10%) with another six minutes stay. Then, 800-μl NaOH (4%) was added and fixed in 5-ml methanol with a thorough blending. After staying at RT for 15 min, the absorbance values were measured at 416 nm, and the standard curve was plotted using rutin standards to calculate the total flavonoid content of each sample. At least three biological replicates were determined.

The Agilent UPLC 1290II-G6400 triple quadrupole mass spectrometer (QQQ; Agilent Technologies, Santa Clara, CA, United States) was employed to determine the relative quantity of synthetic constituents. MS/MS spectra were obtained in negative ionization mode using a C18 column (Eclipse Plus C18, 2.1 × 100 mm, 1.8 μm). The mobile phases were ammonium acetate (A) and acetonitrile (B) solutions with a linear gradient program: 0/5, 2/5, 2.5/18.5, 10.5/41, 11/59, 18/77, 22/95, 24/95, 24.1/5, and 26/5 (min/B%).

### Phylogenetic analysis of *CsFLS* and structural analysis of gene and protein

The *CsFLS* phylogenetic tree was constructed based on the NJ method by obtaining amino acid sequences from various plant FLSs from the GenBank database. Bootstrap tests with 1000 replicates were performed using the MEGA 6.0 software [53].

TBtools software was used to investigate the chromosomal location of *CsFLS* genes and their distribution of exons and introns. Motif conserved motifs (motif parameter set to 10) were predicted using the MEME [54].

### Molecular docking

Swiss-Model [55] was used to model the homologic 3D structures of CsFLS2 and CsFLS3 protein. CsFLS2 and CsFLS3 were modeled based on the anthocyanidin synthase from *A. thaliana* (PDB ID: 1GP4) [56] as the template, and the similarities were 44.55% and 78.10%, respectively. Molecular docking of CsFLS2 and CsFLS3 protein models with their three substrates was performed separately using AutodockTools software, with the substrates DHQ (ChEBI: 17948), DHK (ChEBI: 15404), and Nar (ChEBI:50202) data obtained from CHEBI [57]. In this study, flexible docking was used, and the results were analyzed and plotted by using the PyMOL software.

### Gene cloning and purification and enzymatic activities of recombinant proteins in CsFLS

CsFLS2 and CsFLS3 were cloned using the cDNA as the template and then constructed into a pET28a (+) expression vector. The primers are shown in Additional file 8: Table S5. The constructed recombinant plasmids were transferred into the *E. coli* BL21 (DE3) strain and incubated at 37 ℃, 160 rpm for 2–3 h until OD 600 reached 0.6. The final concentration of 0.4-mM isopropylthio-β-galactoside was added and induced at 16 ℃, 130 rpm for 20 h. After sonication and centrifugation, it was purified through a nickel column and eluted with 250-mM imidazole. N/C-terminal fusion proteins with two His-6 tags were obtained from the condensed elution. After protein concentrations were determined using Bradford reagents (DQ101, Transgen, Beijing, CN), SDS–PAGE electrophoresis and subsequent Western blotting probed using mouse monoclonal antibody Anti-His (30401ES10, Yeasen, Shanghai, CN) were performed. Enzyme reactions with recombinant FLS in a 500 μL system contained 20-mM Tris–Hcl (PH7.0), 1-mg ascorbic acid, 0.1-mg/ml bovine serum albumin, 50-μM ferrous sulfate, 1.5-mg/ml 2-ketoglutarate, 20-μg/ml substrate, and 30-μg recombinant protein reaction at 30 ℃ for 20 min followed by twice extraction using 500-μL ethyl acetate and evaporated dry at low temperature. The reactant was dissolved in 500-μL methanol and filtered for further Q-TOF analysis using the mobile phases of ammonium acetate (A) and acetonitrile (B) and a linear gradient program of 0/5, 2/5, 2.5/18.5, 20/30, 20.5/95, 23/95, 23.5/5, and 26/5 (min/B%). An Agilent 6460 (Agilent, USA) triple quadrupole liquid mass spectrometer in the negative ion mode was used to perform the test as per the parameters set as follows: the scan range setting: 100–1000 m/z, atomization pressure: 35 psi, drying gas flow rate: 8 L min^−1^, protective gas flow rate: 11 L min^−1^, and protective gas temperature: 350 °C. At least three replicates were performed for each sample.

### CsFLS protein activity assays

The substrate concentration was changed to 50 μg/mL, and other conditions were unchanged. The catalytic products were quantified using HPLC with the corresponding standards, and at least three biological replicates were performed.

The optimum pH was determined by conducting the enzymatic reaction at 30 ℃ for 30 min in three buffers (pH 5.0–5.5, 10-mM sodium acetate buffer; pH 6.0–7.5, 10-mM sodium phosphate buffer; pH 8.0–8.5, 10-mM Tris–HCl buffer) at 0.5 intervals. Meanwhile, the optimal temperature was determined by performing the catalytic reaction in pH 7.0 at a 15–50 ℃ gradient range with 5 ℃ interval for every 30 min. The reaction was conducted under the optimum conditions verified above with a substrate concentration range of 0–300 μM. The reactant was detected using HPLC with a linear gradient program of 0/5, 2/5, 2.5/18, 20/30, 20.5/95, 23/95, 23.5/5, and 26/5 (min/B%). The data was stimulated using. The corresponding enzyme kinetic parameters, such as Vmax and Kmax, were calculated by non-linearly fitting the Michaelis–Menten in the Graphpad software. The experiment was repeated at least thrice.

## Supplementary Information

**Additional file 1: Table S1.** Physical characteristics of the major enzyme-encoding genes of flavonoid metabolic pathway in *C. sativa*.

**Additional file 2: Table S2.** Quantitative primers of the selected enzyme-encoding genes of flavonoid metabolic pathway in *C. sativa*.

**Additional file 3: Figure S1.** Analysis of chromosomal location and gene structure of the *CsFLS* genes in *C. sativa*.

**Additional file 4:Table S3.** Secondary structure predication of the CsFLS proteins in *C. sativa*.

**Additional file 5: Table S4.** The list of flavonoid-related genes in the phylogenetic analysis from *C. sativa* and other plant species.

**Additional file 6: Figure S2.** Western blotting of recombinant protein of CsFLS2 and CsFLS3.

**Additional file 7: Figure S3.** Comparsion of CsFLS2 and CsFLS3 with other proteins belonging to DOXC 28/47 subgroup of 2-ODD superfamily.

**Additional file 8: Table S5.** Cloning primers of *CsFLS2* and *CsFLS3* in *C. sativa*.

**Additional file 9: Figure S4.** Cis-acting elements within the promoters of CsFLS2 and CsFLS3.

## Data Availability

The datasets presented in this study can be found in online repositories. The names of repositories and accesion numbers can be found in this manuscript and the additional files.

## Abbreviations

*C. sativa*: *Cannabis sativa*
PAL: Phenylalanine ammonia lyase
C4H: Cinnamate 4-hydroxylase
4CL: 4-coumaric acid: CoA ligase
CHS: Chalcone synthase
CHI: Chalcone isomerase
F3H: Flavanone 3-hydroxylase
FNS: Flavone synthase
F3′H: Flavonoid 3′-hydroxylase
F3′5′H: Flavonoid 3′5′-hydroxylase
FLS: Flavonol synthase
OMT: O-methyltransferase
PT: Prenyltransferase
K: Kaempferol
Q: Quercetin
DHQ: Dihydroquercetin
DHK: Dihydrokaempferol
Nar: Naringenin
Api: Apigenin
M: Myricetin
2-ODD: 2-Oxoglutarate and Fe(II)-dependent dioxygenases
ANS: Anthocyanidin synthase
