# Supplementary figures and images for: Genome-wide identification of key enzyme-encoding genes and the catalytic roles of two 2-oxoglutarate-dependent dioxygenase involved in flavonoid biosynthesis in Cannabis sativa L
Source: Microb Cell Fact. 2022 Oct 15;21:215. doi: 10.1186/s12934-022-01933-y (PMC9571422; doi:10.1186/s12934-022-01933-y)

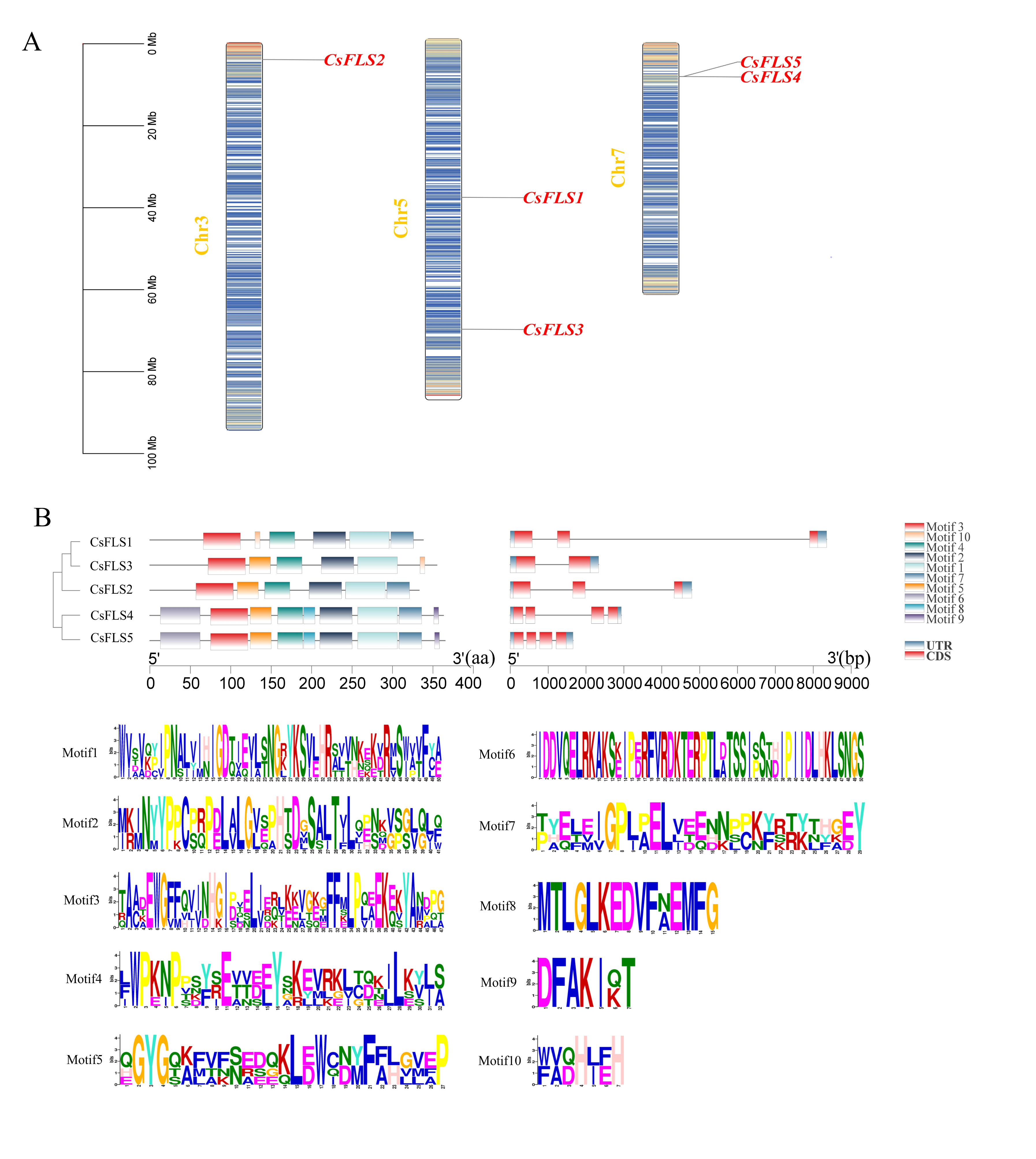

Supplement: Supplementary file 3 — Additional file 3: Figure S1. Analysis of chromosomal location and gene structure of the CsFLS genes in C. sativa. [file 12934_2022_1933_MOESM3_ESM.png]

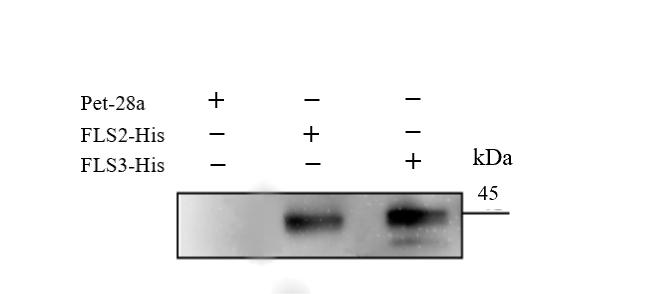

Supplement: Supplementary file 6 — Additional file 6: Figure S2. Western blotting of recombinant protein of CsFLS2 and CsFLS3. [file 12934_2022_1933_MOESM6_ESM.png]

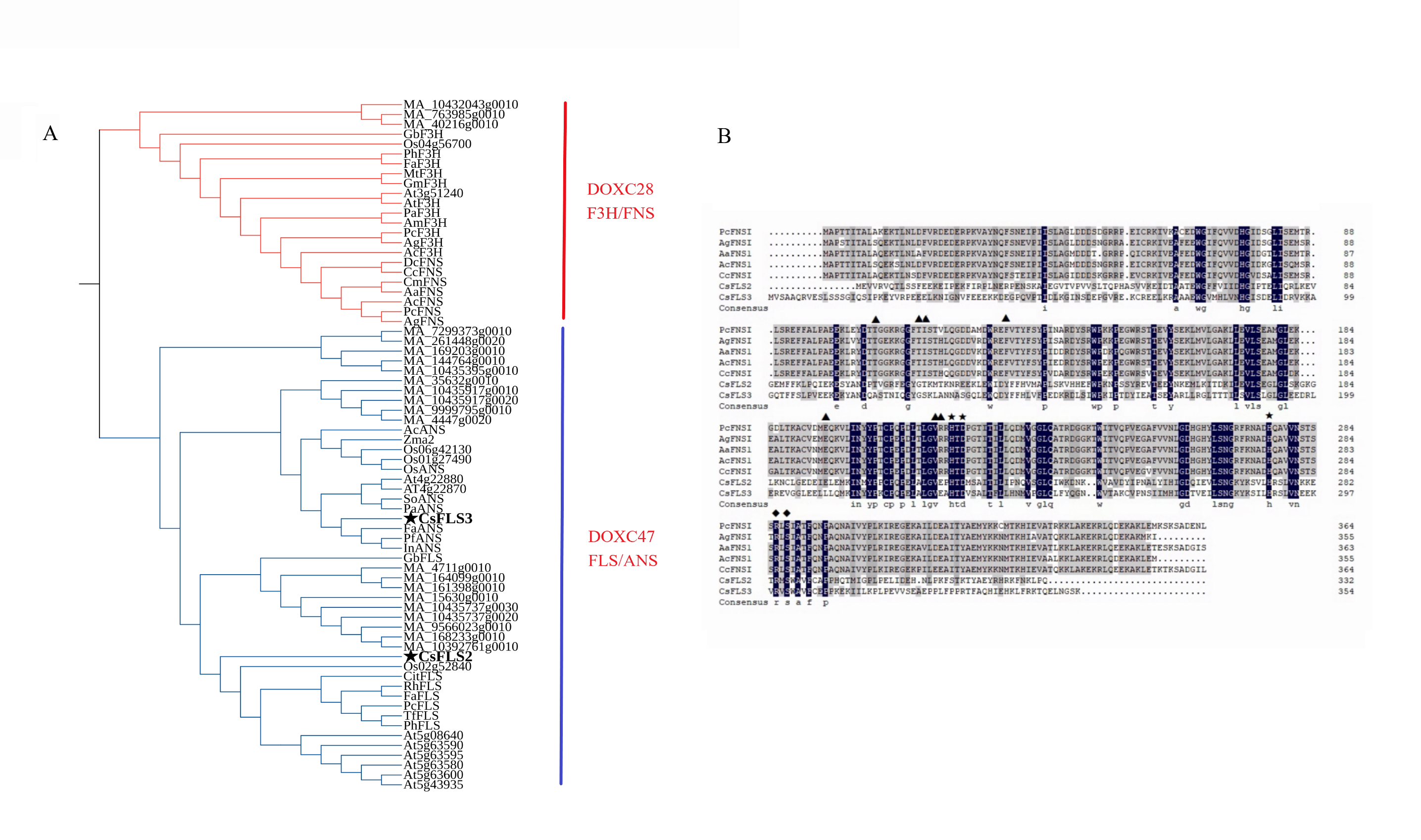

Supplement: Supplementary file 7 — Additional file 7: Figure S3. Comparsion of CsFLS2 and CsFLS3 with other proteins belonging to DOXC 28/47 subgroup of 2-ODD superfamily. [file 12934_2022_1933_MOESM7_ESM.png]

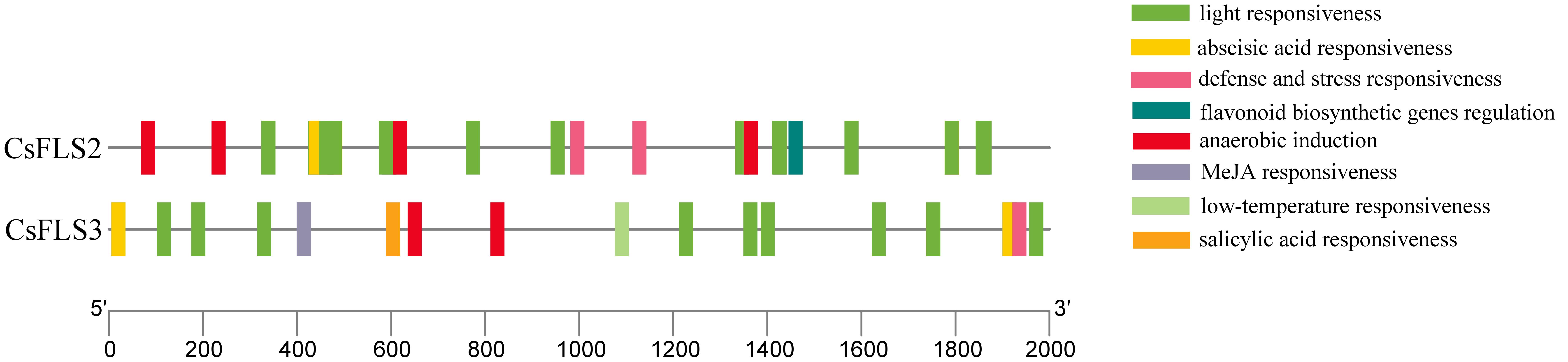

Supplement: Supplementary file 9 — Additional file 9: Figure S4. Cis-acting elements within the promoters of CsFLS2 and CsFLS3. [file 12934_2022_1933_MOESM9_ESM.jpg]
